# Clinical utility of 140-degree scan angle wide-view ultrasound for comprehensive liver and spleen imaging

**DOI:** 10.1007/s40477-026-01117-z

**Published:** 2026-02-10

**Authors:** Seung-seob Kim, Jong Keon Jang, Dong Ho Lee

**Affiliations:** 1 Department of Radiology, Research Institute of Radiological Science, Yonsei University College of Medicine, Seoul, Korea; 2 Department of Radiology and Research Institute of Radiology, Asan Medical Center, University of Ulsan College of Medicine, Seoul, Korea; 3 Department of Radiology, Seoul National University Hospital, 101 Daehak-ro, Jongno-gu, Seoul, 03080 Korea; 4 Department of Radiology, College of Medicine, Seoul National University, Seoul, Korea

**Keywords:** Ultrasonograhphy, Liver, Spleen, Diagnostic imaging

## Abstract

**Purpose:**

Abdominal ultrasound (US) is widely used but often limited by restricted field of view. This study aimed to evaluate hepatic and splenic visualization using wide-view US with a 140° scan angle compared with the routine-view technique in patients undergoing abdominal US.

**Methods:**

In this retrospective study, 258 patients who underwent scheduled abdominal US were included. Each patient received two scans, routine view (70°) and wide view (140°), performed by two experienced radiologists. Two readers independently assigned LI-RADS visualization scores (VIS-A, no or minimal limitations; VIS-B, moderate limitations; and VIS-C, severe limitations) and assessed whether the left lateral segment tip, segment VI tip, and right diaphragm were clearly visualized. Spleen length was measured on both US scans and, when available, on CT or MR images obtained within one year. Proportions were compared using the McNemar test, and agreement of spleen size between US and CT/MR was assessed using the concordance correlation coefficient.

**Results:**

Wide-view US yielded a higher proportion of visualization score VIS-A than routine-view imaging for both readers (81.0–86.8% vs. 67.4–71.7%; *P* < 0.001). Coverage of the left lateral segment tip (96.9–98.4% vs 67.8–77.5%) and segment VI tip (89.5–97.3% vs 75.6–84.1%) was also higher on wide view (*P* < .001). The right diaphragm was more clearly visualized on wide view (89.1–94.6% vs 59.7–69.8%; *P* < .001). Agreement of spleen size with CT/MR was higher for wide-view than routine-view imaging (0.831 vs 0.772).

**Conclusion:**

Wide-view US significantly improved hepatic visualization and spleen measurement accuracy, enabling more comprehensive evaluation.

## Introduction

Abdominal ultrasound (US) is an essential non-invasive imaging modality for evaluating abdominal pain and disease owing to its safety, accessibility, and real-time imaging capability. In the assessment of liver disease, US plays a pivotal role and is often performed before cross-sectional imaging such as CT or MRI in routine clinical practice. Furthermore, since its survival benefit was demonstrated in randomized controlled trials [1, 2], US has been widely adopted for hepatocellular carcinoma (HCC) surveillance in high-risk patients. Many international guidelines recommend liver US every six months for patients at high risk of developing HCC, with or without concomitant serum alpha-fetoprotein testing. A meta-analysis showed a pooled sensitivity of 84% for HCC detection at any stage, with 78% sensitivity in the surveillance setting [3, 4] which may be considered suboptimal. The diagnostic performance of B-mode US in detecting focal liver lesions can be influenced by extrinsic factors such as interposed bowel gas or ribs, which may hinder adequate liver visualization. In particular, the hepatic dome and subdiaphragmatic regions are well-recognized blind spots during liver US examination [5].

Recent advances in ultrasound technology have introduced wide-view scanning, which provides a field of view of up to 140 degrees while maintaining image quality in the lateral regions comparable to that in the central area [6, 7]. By expanding the visual range, this approach may enable more comprehensive evaluation of target organs. In addition, Gao et al. reported that wide-view ultrasound scanning enables accurate liver size measurement with good intra- and inter-operator reliability [6]. In the context of liver US, the wide-view scan has the potential to minimize blind spots and improve overall hepatic visualization, thereby enhancing the detection of focal liver lesions. However, its effectiveness in liver US has not yet been systematically evaluated. Therefore, the aim of this study was to assess the degree of hepatic visualization achieved with the wide-view scan and to compare it with that of the conventional view scan.

## Materials and methods

### Patients

This study was approved by institutional review boards (IRB) of Seoul national university hospital and Severance hospital (Approval No. H-2305–082-1431) and conducted in accordance to the ethical guidelines of the World Medical Association Declaration of Helsinki. The written informed consent was waived by IRB due to retrospective nature of the study. Between January 2023 and February 2023, consecutive patients at two tertiary hospitals who underwent scheduled upper abdominal US for various clinical reasons. All patients were required to fast for at least 6 h prior to US examination. Additional abdominal CT or MR exams were performed if clinically needed.

### US imaging

**Fig. 1:**
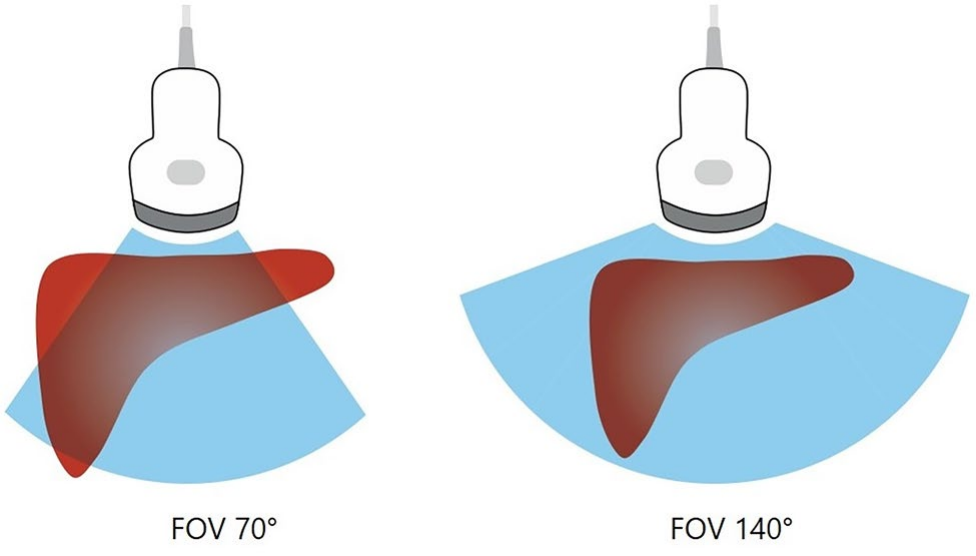
Schematic illustration of routine view and wide view

Two experienced abdominal radiologists (with 17 years and 12 years of experience in US liver imaging, respectively) assessed the liver using a US scanner (Aplio i800, Canon Medical Systems) equipped with a 1–8-MHz convex transducer. Patients were examined in both the supine and left lateral decubitus positions with the right arm raised above the head to optimize liver exposure. Each patient underwent two liver scans: one using the routine view and the other using the wide view (Fig. 1). Both operators followed the standard liver ultrasound scanning protocol recommended by the Korean Society of Ultrasound in Medicine to minimize variability between operators and between routine and wide-view scans [8]. According to the guidelines, essential images include transverse and longitudinal scans covering both hemi-livers from edge to edge and an intercostal scan of the right lobe [8]. For the right hepatic lobe, subcostal scans were performed to visualize the hepatic veins and liver dome, while intercostal scans were used to cover liver segments VIII to VI. As part of the US examination, the spleen was also scanned, and its longest diameter was measured using both the routine and wide-view scans.

### Evaluation of liver visualization

The liver US images were assessed by two experienced abdominal radiologists who conducted the examinations in two separate sessions. To minimize recall bias, image evaluations were performed at least six months after the original examinations, with a minimum interval of two months between sessions. The order of cases was randomized.

Each radiologist assessed the routine-view and wide-view scans separately and assigned a LI-RADS visualization score to evaluate the degree of hepatic visualization [9]. Visualization score VIS-A indicated no or minimal limitations in visualizing the entire liver, score VIS-B indicated moderate limitations (obscuring < 50% of the liver), and score VIS-C indicated severe limitations (obscuring ≥ 50% of the liver). Readers were instructed to assess only the degree of liver obscuration, disregarding intrinsic parenchymal heterogeneity. Reviewers also evaluated whether the left lateral segment tip and segment VI tip, well-recognized blind spots in liver US, were included in each scan. The left lateral segment tip was defined as the lateral margin of the left hepatic lobe at the level where the heart is visualized, and the segment VI tip was defined as the inferior margin of the right hepatic lobe at the level where the right kidney is visible. Image quality of intercostal scans was assessed based on right diaphragmatic visualization. When the entire right diaphragm was clearly depicted on the intercostal view, the scan was classified as optimal.

### Spleen size measurement

During each abdominal US examination, the maximal longitudinal diameter of the spleen was measured on both the routine-view and wide-view scans. For patients who underwent abdominal CT or MR within one year of the US examination, spleen length was also measured on CT or MR images by the same reviewer who evaluated liver visualization, blinded to the US findings. The CT or MR measurement was used as the reference standard for spleen size comparison. To determine the maximal spleen dimension, measurements were obtained on both axial and coronal images, and the largest value was selected for analysis.

### Statistical analysis

The proportions of LI-RADS visualization scores (VIS-A vs. VIS-B or C), coverage rates of the left lateral segment and segment VI tips, and the frequency of optimal right diaphragmatic visualization on intercostal scan between the routine and wide views were compared using the McNemar test. Agreement of spleen size measurements between US and CT/MR examinations was assessed using the concordance correlation coefficient. All statistical analyses were performed with commercially available software (MedCalc, version 20.217; MedCalc Software, Ostend, Belgium).

## Results

### Characteristics

From January to February 2023, a total of 268 upper abdominal US examinations including liver assessment were performed at two university-affiliated tertiary referral hospitals. Ten examinations were excluded because of missing routine-view liver images. The final study population comprised 258 patients (105 men and 153 women; median age, 60.0 years; interquartile range [IQR], 50.0–67.0 years). The indications for upper abdominal US were as follows: evaluation of liver disease (*n* = 163), assessment of gallbladder lesions (*n* = 14), follow-up of pancreatic cysts (*n* = 19), surveillance for metastatic disease in patients with a history of malignancy (*n* = 60), and follow-up for splenomegaly (*n* = 2) (Table 1).

**Table 1:** Patient characteristics

Variables	Value
Age	60.0 (50.0–67.0)
Sex	
Male	105 (40.7%)
Female	153 (59.3%)
Indication of ultrasound examination	
Evaluation of liver disease	163 (63.2%)
Chronic hepatitis B viral infection	66 (25.6%)
Chronic hepatitis C viral infection	6 (2.3%)
Alcoholic liver disease	11 (4.3%)
Fatty liver disease	40 (15.5%)
Primary biliary cirrhosis	6 (2.3%)
Autoimmune hepatitis	7 (2.7%)
Cardiac cirrhosis	5 (1.9%)
Wilson disease	1 (0.4%)
Follow-up for hepatic hemangioma	5 (1.9%)
Evaluation of abnormal liver function test	16 (6.3%)
Evaluation of gallbladder	14 (5.4%)
Follow-up for pancreatic cyst	19 (7.4%)
Surveillance of metastasis	60 (23.2%)
Follow-up for splenomegaly	2 (0.8%)

Note: Continuous variable was presented as median (interquartile range)

### Evaluation of liver visualization

The results of LI-RADS visualization score assignment by each reader, stratified by the presence of liver disease, are summarized in Table 2. For both readers, the proportion of examinations assigned a VIS-A score was higher with wide-view images than with routine-view images, regardless of liver disease status (all *P* < 0.001).

**Table 2:** LI-RADS visualization score on routine view and wide scan view

		Routine view		Wide scan view		*P*-value*
Reader 1	Total (*n* = 258)	Score VIS-A	174 (67.4%)	Score VIS-A	209 (81.0%)	< 0.001
		Score VIS-B/C	84 (32.6%)	Score VIS-B/C	49 (19.0%)	
	Liver disease (*n* = 163)	Score VIS-A	104 (63.8%)	Score VIS-A	125 (76.7%)	< 0.001
		Score VIS-B/C	59 (36.2%)	Score VIS-B/C	38 (23.3%)	
	Other than liver disease (*n* = 95)	Score VIS-A	70 (73.7%)	Score VIS-A	84 (88.4%)	< 0.001
		Score VIS-B/C	25 (26.3%)	Score VIS-B/C	11 (11.6%)	
Reader 2	Total (*n* = 258)	Score VIS-A	185 (71.7%)	Score VIS-A	224 (86.8%)	< 0.001
		Score VIS-B/C	73 (18.3%)	Score VIS-B/C	34 (13.2%)	
	Liver disease (*n* = 163)	Score VIS-A	107 (65.6%)	Score VIS-A	135 (82.8%)	< 0.001
		Score VIS-B/C	56 (34.4%)	Score VIS-B/C	28 (17.2%)	
	Other than liver disease (*n* = 95)	Score VIS-A	78 (82.1%)	Score VIS-A	89 (93.7%)	< 0.001
		Score VIS-B/C	17 (17.9%)	Score VIS-B/C	6 (6.3%)	

Note: *P-value was calculated by McNemar test

**Fig. 2:**
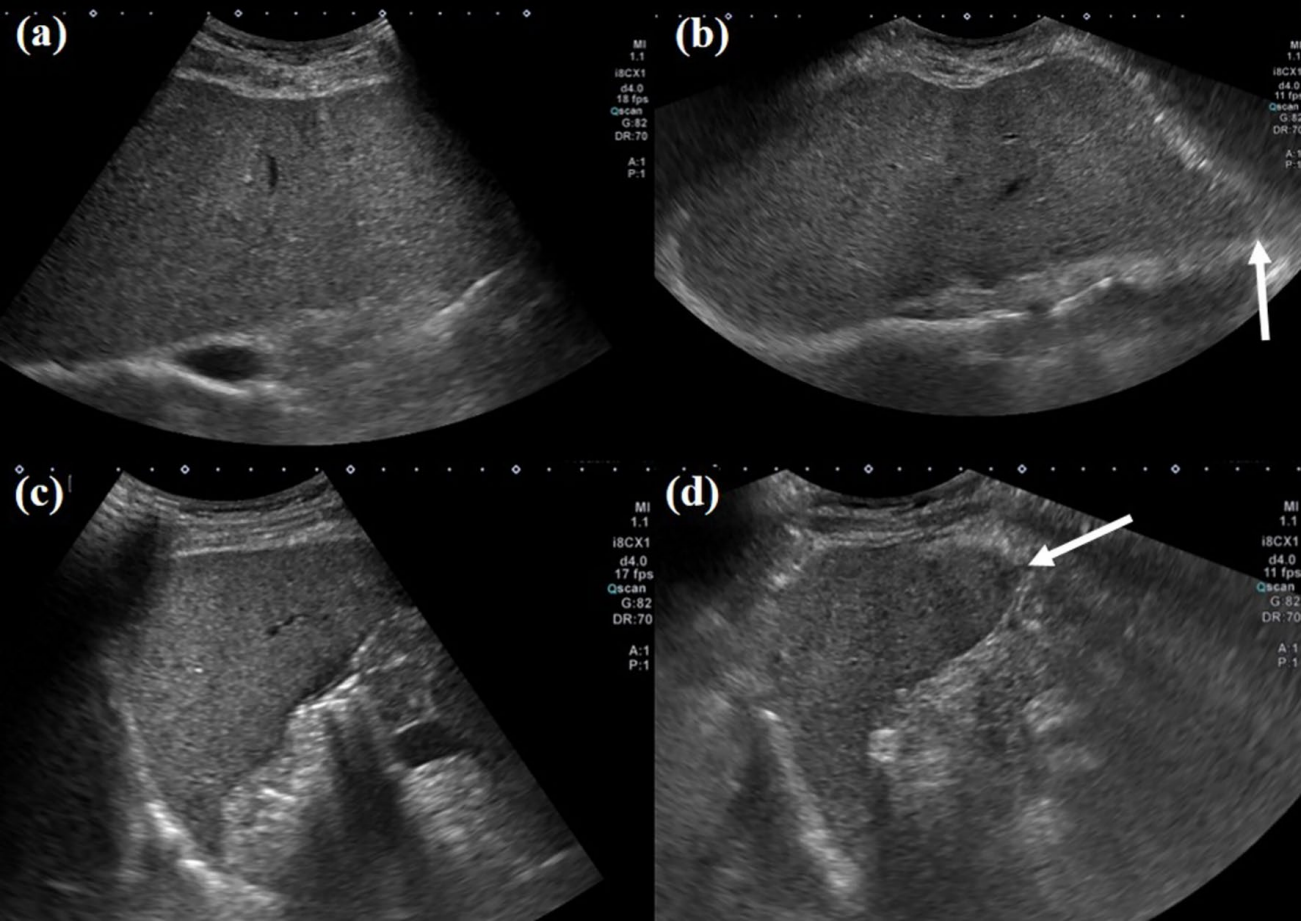
Figure 1. Ultrasound (US) images acquired from a 61-year-old male patient with a history of prior gastric cancer surgery. **a** Transverse liver left lobe scan using a routine view mode, showing limited coverage that does not include the left lateral segment tip of the liver. **b** Transverse liver left lobe scan using a wide view scan mode, providing complete coverage of the border of the liver left lobe, including the left lateral segment tip (arrow). **c** Sagittal liver left lobe scan using a routine view mode, not displaying the left lateral segment tip of the liver. **d** Sagittal liver left lobe scan using a wide view scan mode, including coverage of the left lateral segment tip of the liver (arrow)

**Fig. 3:**
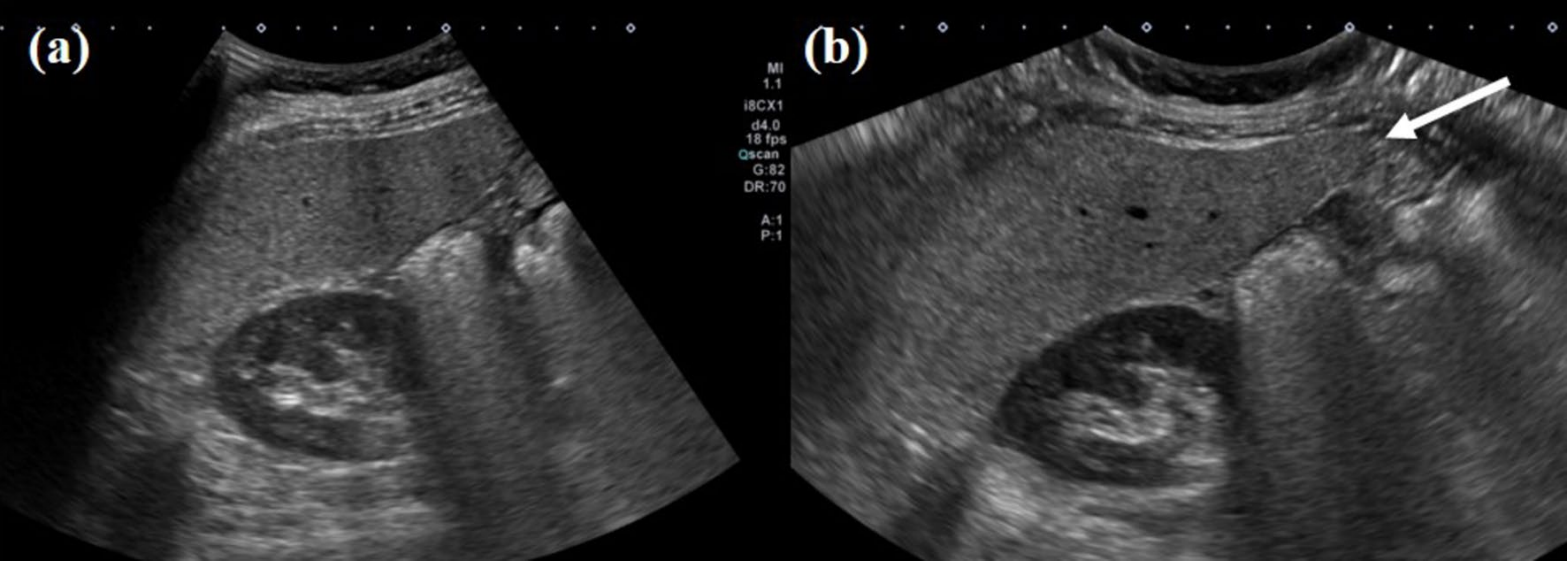
Ultrasound (US) images acquired from a 62-year-old female patient with fatty liver. **a** Intercostal scan of the liver right lobe using a routine view mode, demonstrating limited coverage that does not include the segment VI tip of the liver. **b** Intercostal scan of the liver right lobe using a wide view scan mode, providing complete coverage of the segment VI tip of the liver (arrow)

**Fig. 4:**
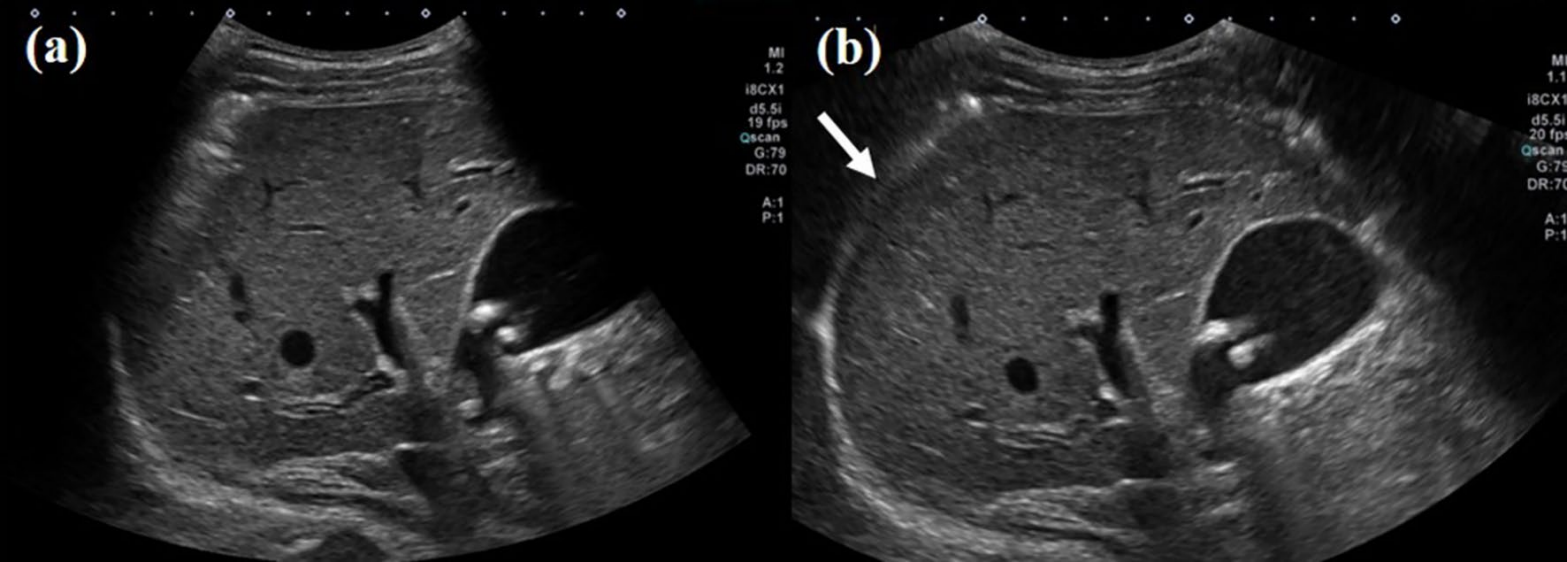
Ultrasound (US) images obtained from a 67-year-old male patient with chronic hepatitis B viral infection. **a** Intercostal scan of the liver right lobe using a routine view mode, displaying an incomplete view of the diaphragm with the subdiaphragmatic area obscured due to the limited field of view. **b** Intercostal scan of the liver right lobe using a wide view scan mode, providing a comprehensive view of the diaphragm edge, and clear visualization of the sub-diaphragmatic area (arrow)

The rates of coverage of the liver left lateral segment tip, segment VI tip, and optimal visualization of the right diaphragm on intercostal views for both wide-view and routine-view imaging, as assessed by both readers, are summarized in Table 3. Wide-view scans demonstrated significantly higher rates of covering the left lateral segment tip (Fig. 2) and segment VI tip (Fig. 3), as well as superior visualization of the right diaphragm (Fig. 4), compared with routine-view scans for both readers (all *P* < 0.001). Overall, wide-view US consistently improved hepatic visualization and reduced blind spots compared with the routine view across all readers and clinical subgroups.

**Table 3:** Coverage of liver tip and right diaphragm visualization on routine view and wide scan view

		Routine view	Wide scan view	*P*-value*
Reader 1	Coverage of left lateral segment tip	77.5% (200/258)	96.9% (250/258)	< 0.001
	Coverage of segment VI tip	75.6% (195/258)	89.5% (231/258)	< 0.001
	Optimal right diaphragm visualization	69.8% (180/258)	89.1% (230/258)	< 0.001
Reader 2	Coverage of left lateral segment tip	67.8% (175/258)	98.4% (254/258)	< 0.001
	Coverage of segment VI tip	84.1% (217/258)	97.3% (251/258)	< 0.001
	Optimal right diaphragm visualization	59.7% (154/258)	94.6% (244/258)	< 0.001

Note: *P-value was calculated by McNemar test

### Measurement of spleen size

**Fig. 5:**
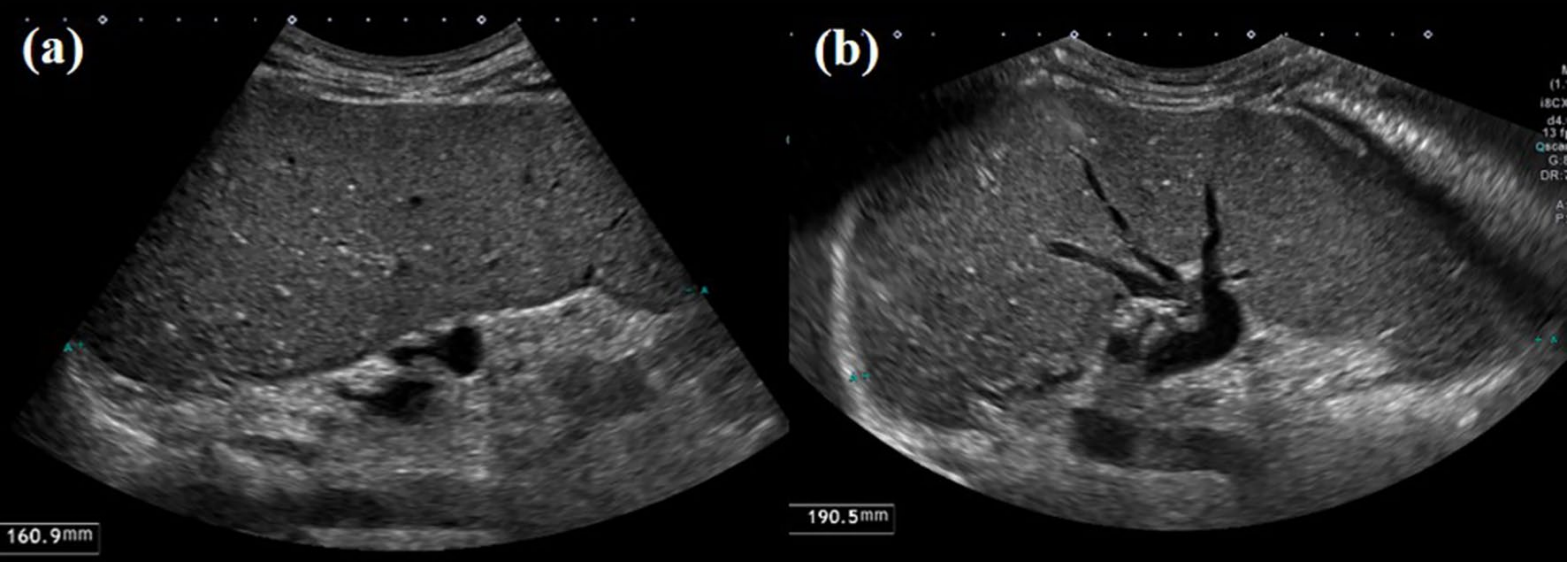
Ultrasound (US) images obtained from a 62-year-old female patient with liver cirrhosis related to hepatitis B viral infection (HBV). **a** In routine view mode, the limited field of view does not cover the full length of the spleen, resulting in a measured spleen size of 16.1 cm. **b** Using wide view scan mode, the entire length of the spleen is displayed, resulting in a measured spleen size of 19.1 cm. A CT scan performed six months prior to the US examination measured the spleen size at 18.9 cm

Of the 258 patients included, 131 (50.8%) underwent abdominal CT or MR examinations within one year of the US study. The median spleen size was 9.9 cm (IQR, 9.0–11.1 cm) on CT/MR, 8.8 cm (IQR, 7.7–10.0 cm) on routine-view US, and 9.1 cm (IQR, 7.8–10.5 cm) on wide-view US. The concordance correlation coefficient for spleen size was 0.772 (95% confidence interval [CI]: 0.707, 0.824) between CT/MR and routine-view US, and 0.831 (95% CI: 0.778, 0.873) between CT/MR and wide-view US (Fig. 5). Wide-view scan demonstrated a stronger agreement with CT/MR measurements than the routine view, indicating improved accuracy in spleen size assessment.

## Discussion

In this study, we evaluated the effectiveness of wide-view US imaging of the liver in patients undergoing abdominal US examinations. Wide-view scan resulted in a significantly higher proportion of patients with a LI-RADS visualization score VIS-A compared with the routine view in the overall cohort (81–87% vs 67–72%; *P* < 0.001) and among patients with liver disease (77–83% vs 64–66%; *P* < 0.001). Coverage of the hepatic tips was also greater with wide-view imaging (90–98% vs 68–84%; *P* < 0.001), and the right diaphragm was more clearly visualized on intercostal scans (89–95% vs 60–70%; *P* < 0.001). Overall, wide-view US enabled a more comprehensive evaluation of the liver by reducing common blind spots seen on routine-view imaging, particularly in the liver tip and subdiaphragmatic regions.

Full visualization of the liver on US is a prerequisite for high-quality HCC surveillance. Previous studies have shown that a LI-RADS visualization score of VIS-B or C is associated with a higher likelihood of false-negative HCC detection compared with score VIS-A [8, 10]. In the present study, wide-view imaging yielded a significantly higher proportion of visualization score VIS-A than routine-view imaging in paired examinations of the same patients, irrespective of operator or underlying liver disease (81–87% vs 67–72%; *P* < 0.001). The improved hepatic visualization achieved with wide-view imaging may be attributed to its broader acoustic beam (140° vs 70°), which extends the field of view and enables clearer depiction of the hepatic tips and diaphragm. This advantage of wide-view imaging may be particularly relevant in patients with viral cirrhosis, in whom right-to-left liver volume redistribution often limits complete visualization on standard [11]. Similarly, wide-view imaging could provide improved surveillance performance in patients with compensatory hypertrophy of one hepatic lobe after partial hepatectomy or following portal vein embolization performed to increase the future liver remnant before resection. In addition, wide-view imaging consistently provided superior visualization of the left lateral segment tip, segment VI tip, and right diaphragm on intercostal views compared with routine-view imaging, suggesting a reduction in blind spots during liver US examination. However, as this study evaluated only a 140° wide-view configuration provided by the manufacturer, further studies assessing various angular settings are warranted to determine the optimal field of view for liver imaging.

Measurement of spleen size is an integral component of abdominal US, as both splenomegaly and splenic atrophy can indicate various underlying diseases. On US, the longest anteroposterior diameter of the spleen is typically measured, and this parameter has been shown to correlate well with the actual splenic length observed at autopsy [12]. However, a previous study reported only moderate interobserver agreement for spleen size measurement, with an intraclass correlation coefficient of 0.67 [13]. In patients with splenomegaly, accurate measurement of spleen size can be particularly difficult because the long axis of the spleen often does not align with the imaging plane, making full visualization challenging on conventional US. The wide-view technique, which offers an expanded field of view through a broader beam angle, allows more comprehensive visualization of the spleen and may improve measurement accuracy and consistency. In this study, the concordance correlation coefficient for spleen size between US and CT or MR reference standards was higher with the wide view than with the routine view (0.831 vs 0.772), indicating improved measurement accuracy. Consistent with these findings, a recent study by Gao et al. reported that wide-view scans enabled accurate liver size measurements with good intra- and inter-operator reliability [6], supporting the potential utility of wide-view techniques for organ size assessment.

This study has several limitations. First, because of its retrospective design, complete control of selection bias was not possible. In addition to the hepatic coverage area, intrinsic patient factors such as obesity and hepatic steatosis, which are known to influence LI-RADS visualization scores [9], were not controlled during image assessment. Second, although wide-view imaging demonstrated improved visualization based on LI-RADS scores, we did not evaluate its direct clinical impact, such as diagnostic accuracy for HCC detection. Future studies in high-risk populations are warranted to determine the incremental diagnostic value of wide-view US for HCC surveillance. Third, most patients with chronic liver disease in this study had hepatitis-related etiologies; therefore, the findings may not be generalizable to other causes of chronic liver disease. Nevertheless, the proportion of visualization score VIS-A remained consistently high regardless of the presence of chronic liver disease. Fourth, because both wide-view and routine-view scans were obtained in the same session, recall bias during image acquisition could not be completely excluded. However, to minimize this bias, image evaluations were performed at least six months after acquisition, and two independent reading sessions were separated by a minimum two-month interval. Fifth, wide-view imaging is currently available from few manufacturers, which may further limit the generalizability of the study results.

In conclusion, the use of a wide view with 140-degree scan angle significantly improves LI-RADS visualization scores compared to routine scans by increasing the coverage of previously challenging areas, specifically the left lateral tip and sub-diaphragmatic area of the liver. In addition, wide view could visualize the spleen more clearly than routine view. Therefore, wide view could provide a thorough evaluation of the liver and spleen.

## Abbreviations

CT: Computed tomography
HCC: Hepatocellular carcinoma
LI-RADS: Liver imaging reporting and data system
MR: Magnetic resonance
US: Ultrasound
